# The promising application of cell-cell interaction analysis in cancer from single-cell and spatial transcriptomics

**DOI:** 10.1016/j.semcancer.2023.07.001

**Published:** 2023-07-15

**Authors:** Xinyi Wang, Axel A. Almet, Qing Nie

**Affiliations:** a Department of Mathematics, University of California, Irvine, Irvine, CA, United States; b The NSF-Simons Center for Multiscale Cell Fate Research, University of California, Irvine, Irvine, CA, United States; c Department of Developmental and Cell Biology, University of California, Irvine, Irvine, CA, United States

**Keywords:** Single-cell RNA-sequencing, Spatial transcriptomics, Cell-cell interactions, Cancer

## Abstract

Cell-cell interactions instruct cell fate and function. These interactions are hijacked to promote cancer development. Single-cell transcriptomics and spatial transcriptomics have become powerful new tools for researchers to profile the transcriptional landscape of cancer at unparalleled genetic depth. In this review, we discuss the rapidly growing array of computational tools to infer cell-cell interactions from non-spatial single-cell RNA-sequencing and the limited but growing number of methods for spatial transcriptomics data. Downstream analyses of these computational tools and applications to cancer studies are highlighted. We finish by suggesting several directions for further extensions that anticipate the increasing availability of multi-omics cancer data.

## 1. Introduction

Cells coordinate to perform functions as a multicellular organism. Such coordination can be achieved through cell-cell interactions (CCI)—also known as cell-cell communication or cell signaling—where cells communicate with nearby cells by sending and receiving molecular messages. Within multicellular organisms, there are distinct groups of specialized cells with distinct cell functions, which facilitate different types of CCI. These range of interaction behaviors are crucial to many biological processes, including cell growth, division, differentiation, tissue or organ development, and disease progression [1–3]. For example, in the context of cell differentiation, T follicular helper cells, a subset of CD4+ T cells, are found to secrete IL-4 and IL-21 ligands that promote B cell proliferation and B cell differentiation into plasma B cells or germinal center B cells [4]. In embryonic development, WNT signaling are found to play diverse roles in cell fate determination, differentiation, proliferation and apoptosis [5,6]. In disease progression, some ligands and receptors are found to inhibit interactions involved in facilitating the immune responses. For instance, cytotoxic T lymphocyte-associated antigen-4 (CTLA-4) was found to downregulate immune responses and is closely related to tumor progression [2]. In particular, CCI is important for cancer studies. Many cellular processes that are crucial for the development of cancer are regulated by CCI, including cell growth and division, cell apoptosis, cell motility and invasion, angiogenesis, inflammation and immune suppression [7–9].

**Fig. 1.**
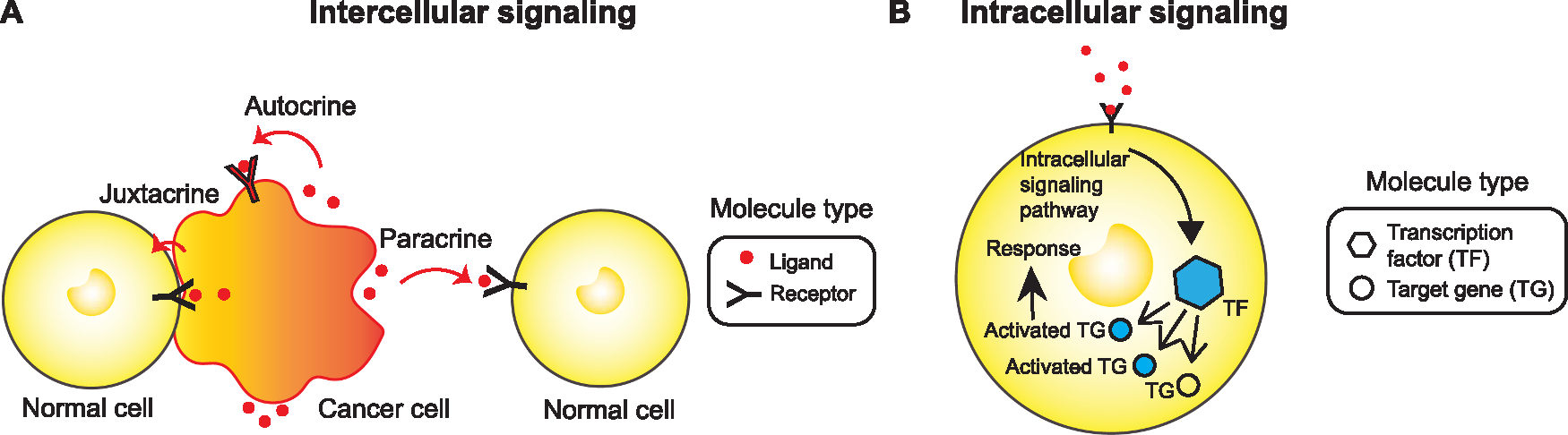
The biological components of cell-cell interactions that can be inferred from single-cell and spatial data. A The different types of intercellular CCI, which include autocrine, paracrine, and juxtacrine signaling. Autocrine signaling occurs when a cell receives the same signals secreted by itself. Paracrine signaling occurs when a cell receives a signal secreted by nearby cells. Juxtacrine signaling occurs a cell receives a signal from a directly adjacent cell through physical contact. B Intracellular CCI is the downstream response to intercellular signaling that takes place inside cells. When a sufficient number of ligands bind to associated receptors, the receptors are activated. The signal received by receptors will be converted into an intracellular signal and activate transcription factors and target genes.

There are two components of CCI. One is intercellular signaling, which occurs between cells and at the cell membrane (Fig. 1A). The other is intracellular signaling, which is the downstream response to intercellular signaling and takes place inside the cells (Fig. 1B). Intercellular signaling consists of sender cells secreting signaling molecules, called ligands, into the extracellular space. Each ligand can then only bind to a certain set of proteins located on the membrane of possible receiver cells called receptors. When a sufficient number of ligands have reached and binded to their appropriate receptors, different transcription factors and consequently target genes within the cell are activated downstream. There are four types of cell communication: autocrine, paracrine, endocrine and juxtacrine. Autocrine signaling is defined as a cell receiving a signal secreted by itself. For example, it was found that autocrine TGF-β signaling maintains self-renewal in human embryonic stem cells [10]. Paracrine signaling is defined as a cell receiving a signal secreted by other nearby cells. For example, paracrine Interleukin-1 signaling from carcinoma cells induces cytokine secretion in mesenchymal stem cells [11]. Juxtacrine signaling requires direct physical contact between cells, where signals are secreted across gap junctions between cells in contact. An example of this type of signaling is the interaction between a membrane-bound ligand Delta and the cell-surface receptor Notch [12]. Signals secreted during endocrine signaling travel a long distance from sender cells through the circulatory system to reach potential receiver cells. The release of hormones from glands and travel through the bloodstream to reach distant body sites is an example of endocrine [13]. However, due to the far spatial distances required for endocrine signaling, scRNA-seq and ST data do not provide enough information to infer endocrine signaling.

**Fig. 2.**
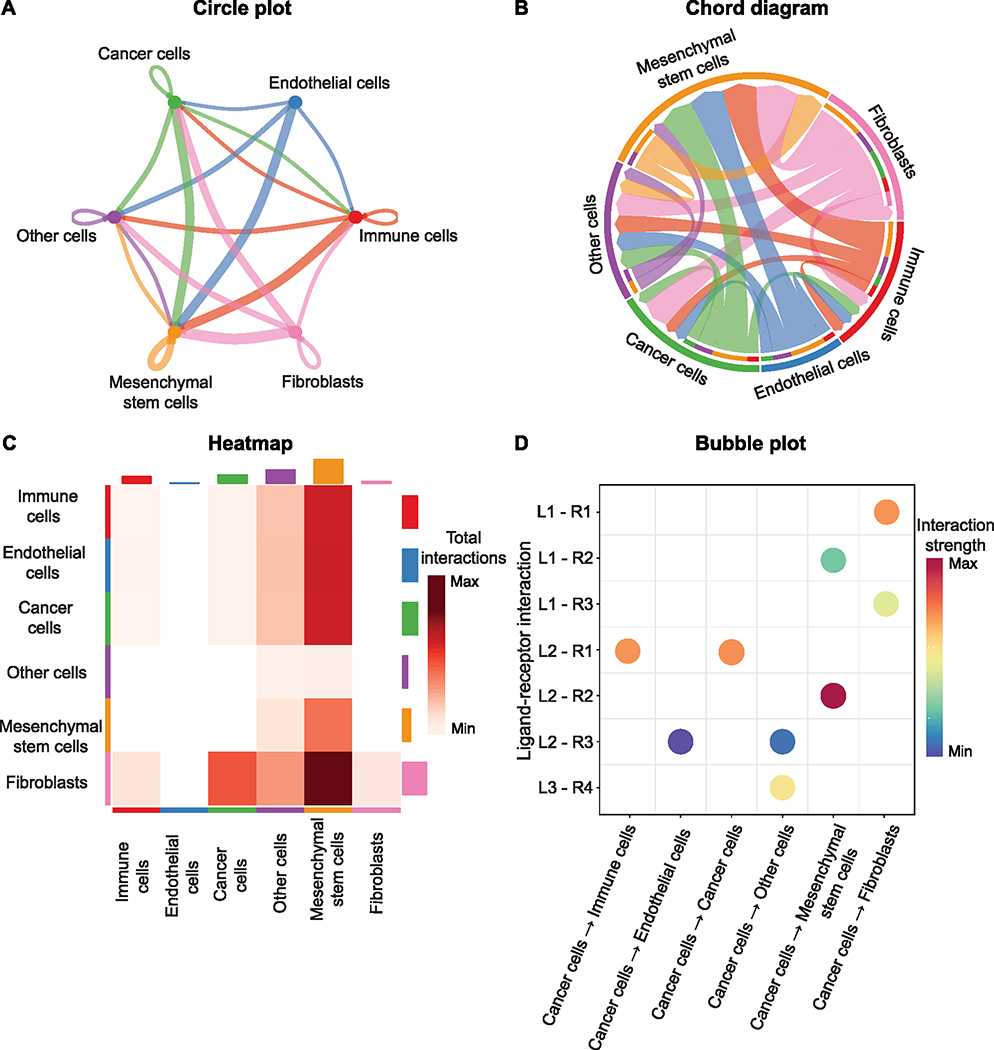
Common visualizations of CCI inference from cancer datasets A Circle plot showing directed interactions between cell types. B Chord diagram indicating outgoing interactions from different cell types. C Heatmap showing the total interaction strength between sender and receiver cell types. D Bubble plot displaying the interaction strength between cancer cells and other cell types with respect to different ligand-receptor pairs. L1, L2, L3 represent three different ligands, and R1, R2, R3, R4 represent four different receptors.

Single-cell RNA sequencing (scRNA-seq) and spatial transcriptomic (ST) technologies are rapidly developing, enabling the profiling of biological tissue at unprecedented genomic depth. The gene expression profile of tens of thousands of genes, many of which are related to CCI, can be captured for thousands and potentially millions of cells [14]. Being able to capture such rich genomic information offers a great opportunity to change how CCI can be investigated in different tissues of different species. For known ligand-receptor interaction pairs, based on the assumption that higher levels of relevant ligand and receptor gene expression reflect a higher possibility of CCI occurring, one can use the expression of ligand and receptor genes in possible sender and receiver cells to infer CCI. A great number of bioinformatics tools have been developed recently to model and analyze CCI between and within cells based on gene expression data obtained from non-spatial single-cell and spatial transcriptomics data—and many more continue to be developed. Using these computational tools, we can infer an approximate landscape of CCI from scRNA-seq data (or ST data) and advance our understanding of CCI mechanisms in different biological systems. Indeed, there is a rapidly growing number of applications where these tools have been used to reveal important and novel CCIs from scRNA-seq studies of cancer [15–18]. Most CCI tools consist of two components: a ligand-receptor database which contains the possible ligand-receptor pairs and a computational model to calculate the likelihood of CCI based on the expression values of the ligand and receptor genes. Some CCI tools, such as CellChat [19], include functionality to visualize the CCI networks directly (Fig. 2). In this paper, we will briefly review the currently available computational tools that model CCI based on gene expression data obtained from non-spatial scRNA-seq or ST data. We discuss current ways to perform downstream analysis after inferring CCI, how CCIs are validated, the applications of CCI results in cancer, and possible future directions.

### 2. A. CCI methods

Many computational CCI tools have been developed based on either non-spatial scRNA-seq data or have used ST data to constrain potential interactions. The CCI tools usually take preprocessed data, which includes normalization by library depth and log-transformation, as input. These tools cover both the calculation of intercellular interactions and intracellular interactions. While these tools have the common goal of modeling CCI, they are based on different computational strategies and biological assumptions. We will describe the currently available tools in detail below.

### 2.1. Nonspatial CCI methods

In this section, we briefly describe the categories of computational methods that CCI tools use to model the different components of cell signaling (Table 1).

### 2.2. Intercellular CCI methods

Intercellular CCI methods are used to infer potential interactions between cells. More specifically, they try to capture the interaction strength, which is assumed to reflect the probability of an interaction occurring, between associated ligands and receptors.

#### Threshold-based methods

One type of CCI method removes insignificant interactions based on individual ligand and receptor expression levels, where ligands and receptors are only considered if their expression levels exceed a predetermined threshold in their respective sender and receiver cell types. After thresholding, only ligand-receptor pairs where both the ligand and receptor are retained for CCI inference. These types of methods output a binary CCI score. One example of this type of method is CellTalker [20], which only considers ligands and receptors with non-zero expression in more than 5% of cells for inference, or ligands and receptors with non-zero expression in more than 3% of certain cell type groups for specific interactions.

#### Differential-expression-based methods

Differential-expression-based CCI methods infer significant interactions by first identifying differentially expressed ligands and receptors using statistical models. The differentially expressed ligands and receptors are then compared to existing ligand-receptor pairs in a curated CCI database. The output of these CCI methods are generally interpreted as a binary score for all ligand-receptor pairs. PyMINEr is an example of one such CCI method [21]. It first finds the significantly enriched ligands and receptors in each cell type based on both analysis of variance (ANOVA) and *z*-score enrichment, and then cross-references ligand-receptor pairs with the StringDB interaction list to only include direct binding pairs. Another example of one such CCI method is iTALK [22], which allows for the use of one of several differential expression methods for single-cell applications to find differentially expressed ligands and receptors.

#### Permutation-based methods

Permutation-based CCI methods measure the specificity of an interaction between two cell types. There are two types of permutation tests considered: tests that permute either gene labels or cell type labels. Gene label permutation tests measure how high the observed ligand (receptor) expression levels are compared with a “null distribution” obtained from randomly selected genes. Cell type label permutation tests measure how high the ligand and receptor expression level in the considered cell types are compared to randomly assigned cell type labels.

One example of a method that performs gene label permutation tests is scSeqComm [23]. Given a fixed cell type, it first randomly resamples genes from all genes and computes the distribution of the average gene expression of a randomly resampled gene. The distribution of average gene expressions observed by chance is then approximated using a Gaussian distribution. The score of the ligand (receptor) in the fixed cell type is computed as the probability of observing lower values from the approximated distribution than the average gene expression of the ligand (receptor) of interest. The ligand-receptor score is calculated as the minimum of the ligand and receptor score.

CellPhoneDB [24] is an example of a method that performs cell type label permutation. Assuming cell type annotations have been provided, CellPhoneDB calculates an enrichment score of ligand-receptor interactions between two cell types based on the gene expression of ligand and receptor in sender and receiver cell types, respectively. This enrichment score is calculated as the minimum of the average gene expression of ligand and receptor in their respective cell types. Next, by randomly permuting the cell type labels of each cell a large number of times, a null distribution of enrichment score of ligand-receptor interaction is calculated. Then the interaction score of a ligand-receptor pair is calculated as the proportion of enrichment scores that are higher than the actual computed enrichment score.

#### Coexpression-based methods

The majority of CCI methods analyzed in this paper are based on the measuring the simultaneous coexpression of ligands and receptors. These types of methods can be further divided into three subcategories based on their calculation formula; that is, whether they are sum based, product based, or correlation based.

##### (i). Sum-based coexpression:

Sum-based coexpression methods infer CCI based on a calculated sum of certain features of ligand and receptor expression. For example, for a given ligand-receptor interaction, CellCall infers intercellular communication by calculating the Euclidean norm of a vector consisting of the normalized ligand and receptor expression values, which is then weighted by an activity score of associated transcription factors [25]. CytoTalk is another method that uses a sum-based method to calculate the ligand-receptor interaction strength [26]; possible interactions are weighted by multiplying the sum of features of ligand and receptor expression with a “non-self-talk” score, which is calculated using the mutual information of ligand and receptor expression in the sender group and receiver groups, respectively.

##### (ii). Product-based coexpression:

Product-based coexpression methods, as their name suggests, infer CCI based on the product of ligand and receptor gene expression. CellChat [27] is one such popular method. CellChat considers the expression levels of ligands and receptors in respective sender and receiver cell groups, respectively. When considering multi-units of ligands or receptors, CellChat uses the geometric mean of each gene to approximate the average expression level.The communication probability between two cell groups for a given ligand-receptor pair is then defined as a product based on mass action kinetics, where the “base” ligand-receptor score, calculated as a normalized product of ligand and receptor expression, which is then weighted by the average expression of known agonists and antagonists.

Product-based coexpression methods are popular for CCI inference. Other product-based methods include: NATMI [28], SingleCellSignalR [29], ICELLNET [30], scConnect [31], CSOmap [32], SoptSC [33], and Connectome [34]. Each method uses its own “product-based” formula to infer ligand-receptor interaction strength.

#### (iii). Correlation-based coexpression:

Correlation-based coexpression methods infer CCI based on statistical correlation between gene expression in cell groups of interest. These methods assume that correlation in expression of two genes correspond to regulation by a common signaling mechanism. REMI calculates the Pearson correlation between ligand and receptor gene expressions to model the likelihood of interaction [35].

### 2.3. Intracellular CCI methods

Intracellular CCI methods model the interaction process within cells. Some intracellular CCI methods specifically model downstream networks containing interactions from receptors to transcription factors, and from transcription factors to downstream target genes, while others model interactions among all possible intracellular genes within the cells.

#### Fisher’s Exact test

To construct the intracellular signaling network, scMLnet utilizes Fisher’s exact test [36] to compute the activity score of a specified transcription factor in a given cell type [37]. First, known associations between receptors and transcription factors and between transcription factors and target genes are curated from existing public databases. Next, three sets are constructed: the set of significantly expressed target genes, the set of all possible target genes, and the set of target genes for a given transcription factor. Using the sizes of the intersection sets between these three constructed gene sets, Fisher’s test is used to compute the *p*-value, which represents the activity of a specific transcription factor in a certain cell type. This *p*-value of a specific transcription factor is lower if there are more highly expressed target genes regulated by it, indicating a higher activity of the transcription factor of interest. Cheng et al. use scMLnet to only consider transcription factors with a calculated *p*-value lower than 0.05 to be activated in the receiver cells, which are then included in the subnetwork describing interactions from transcription factors to target genes [38]. Similarly, to find links between receptors and transcription factors, Cheng et al. also use Fisher’s test is used to compute the activity of receptors according to the calculated *p*-value. Aside from scMLnet, other tools that use Fisher’s test to determine the intracellular interactions are CCCExplorer [39] and scSeqComm [23].

#### Differential expression-based

Another method, CellCall [25], uses gene set enrichment analysis to calculate an activity score of transcription factors that are activated downstream of a ligand-receptor interaction. CellCall first constructs regulons consisting of a transcription factor and its set of coexpressed target genes. The Spearman’s rank correlation coefficient is used to determine gene coexpression between a target gene and a transcription factor; significantly coexpressed genes are retained in the considered regulon. Next, the activity score of the transcription factor is calculated using the gene set enrichment analysis enrichment score for the regulon. If there are multiple transcription factors downstream of a ligand-receptor interaction, then the transcription factor activity score is calculated as the weighted sum of all transcription factors downstream of the interaction.

#### Network-based

Network-based methods use network analysis methods that utilize the structure of the network to infer likely interactions. scSeqComm uses this type of method to build the receptor-TF subnetwork, first by using existing databases. Next, the PageRank algorithm, a network analysis method in machine learning, is used to calculate the strength of associations between receptors and transcription factors, where the receptor as the seed node. The PageRank scores of each transcription factor is used to measure the association of the transcription factor to a given receptor.

#### Coexpression-based

Rather than consider interactions from receptors to transcription factors and from transcription factors to target genes, one intracellular method, CytoTalk, constructs an intracellular gene coexpression network between all possible pairs of genes within cells using mutual information [26].

### 2.4. Ligand-target gene network CCI

Most CCI methods that consider intracellular signaling networks model intercellular CCI and intracellular CCI separately. In contrast, NicheNet constructs a ligand-target network that connects signaling ligands to downstream target, using PageRank, a network-based method [40]. NicheNet uses ligand-receptor networks, signaling networks, and gene regulatory networks constructed using multiple existing databases. These networks contain interactions from ligands to downstream transcription factors, and gene regulatory interactions between transcription factors and target genes. The data sources are integrated to build a weighted ligand-signaling network and gene regulatory network. Based on the ligand-signaling network, for target genes and upstream ligands of interest, a signaling importance score for each ligand to each target gene is calculated using a Personalized PageRank algorithm. Accordingly, a ligand-gene signaling importance matrix is obtained, where each entry denotes the signaling importance of each upstream ligand to each downstream gene. Next, the final ligand-target gene interaction network is obtained by multiplying the ligand-gene signaling importance matrix and the weighted integrated gene regulatory network matrix. The entries in this matrix denote thus the regulatory potential of a ligand to a downstream target gene.

### 2.5. Spatial CCI

Spatial transcriptomic technologies are rapidly expanding, which include immunofluorescence-based methods, mass spectrometry-assisted methods, and barcoding-based methods [41]. In contrast to scRNA-seq data in which the spatial information is destroyed, spatial data preserves not only cell-cell heterogeneity information but also spatial positions. Since CCI can only occur between spatially proximal cells, knowing the cellular positions in space allows one to constrain the prediction of potential ligand-receptor interactions and significantly reduce the prediction of false positive interactions. There are two assumptions that are often used when inferring CCI from spatial data. First, CCI is a result of ligand-receptor co-occurrence. Second, the gene expression of cells also depends on their interactions with neighboring cells. Recent advancement in spatial transcriptomics makes it possible to detect genetic information at multiple cells, single-cell and even subcellular resolutions [42]. These advancements makes it possible to explore the above assumptions. Computational tools incorporating spatial information are developed based on these two different assumptions to capture CCI activity. Tools based on the first assumption include Giotto, SpaOTsc, GCN, and DeepLinc, and another tool based on the second assumption is SVCA. In contrast to most tools developed to analyze spatial data directly, SpaOTsc tries to map non-spatial scRNA-seq, which has single-cell resolution and typically higher gene coverage, to the positions of spatial data, and then analyze CCI using the mapped scRNA-data. We will briefly describe existing CCI methods utilizing spatial information below (Table 2).

Giotto [43] uses spatial information to constrain possible cell interactions and then models CCI strength by calculating a sum-based coexpression score of ligands and receptors. Giotto first determines whether two cell types are preferentially located in a spatially proximal manner and then tries to identify which ligand-receptor pairs interact between two spatially proximal cell types. For each possible ligand-receptor pair in sender cell type and receiver cell type, the interaction score is a sum-based coexpression score, which is calculated by the weighted average gene expression of ligand and receptor in interacting sender and receiver cells, or in the subset of sender and receiver cells that are spatially close. Giotto then uses a permutation test to assess whether the calculated interaction score is statistically significant. Rather than randomly permuting cell type labels, Giotto randomly permutes the cell locations within the same cell type before calculating the corresponding *p*-value.

SpaOTsc is a tool that uses spatial data as a reference to provide spatial information to a non-spatial scRNA-seq dataset, from which ligand-receptor interactions are inferred using optimal transport [44]. First, SpaOTsc constructs a spatial metric for the non-spatial dataset. Using the pairwise gene expression similarity between cells in the non-spatial scRNA-seq dataset and cells in the reference spatial dataset, SpaOTsc assigns a position (as a probability distribution over all spatial positions) to each cell in scRNA-seq dataset using optimal transport. Based on this position assignment, SpaOTsc calculates a spatial metric between each pair of cells in scRNA-seq dataset. Then the genes in scRNA-seq data can be viewed as distributions over all cells in the dataset. For a given ligand-receptor pair, SpaOTsc calculates an optimal transport plan from ligand distributions to receptor distributions, where the cost function is defined as the spatial distances between cells. This transport plan gives the ligand-receptor interaction between each cell pair. SpaOTsc then summarizes the interactions between cells across cell type groups to calculate interactions at the level of cell types.

COMMOT is an extension of the optimal transport framework of SpaOTsc that further considers the competition between multiple ligands and multiple receptors [45]. Instead of viewing CCI between cells as an optimal transport plan between probability distributions of a ligand and a receptor, COMMOT considers CCI between cells as a collection of optimal transport plans for all ligands and receptors that can be coupled simultaneously under some spatial constraint. To consider competition between ligands or receptors, COMMOT assumes that a specific ligand or receptor in a specific cell has limited capacity for interactions that depends on ligand or receptor expression in the specific cells. Thus, in a given pair of cells, for a specific ligand that can bind to different receptors, a stronger interaction with one receptor reduces its potential of interaction with another receptor. The CCI between certain ligand-receptor pair in a pair of cells is given by optimizing a collection of transportation plans. Similar to SpaOTsc, COMMOT then aggregates the interactions between cells in clusters to calculate cluster-level CCI.

GCNG is a spatial CCI method based on supervised learning on graph neural networks [46]. The graph neural network takes a cell neighborhood graph as input. A graph Laplacian is then calculated to encode intercellular spatial relationships in the graph structure, and specific ligand-receptor pair expression in each cell are specified as node attributes. The output of the graph neural network is a binary value which represents whether the specific ligand-receptor interaction exists in this graph.

DeepLinc is a spatial method that uses a variational graph autoencoder (VGAE) to infer CCI [47]. A VGAE takes a cell neighborhood graph, where edges connect neighboring cells, and gene expression as features of the nodes in the graph as input. The encoder of VGAE outputs a latent representation for each cell. Next, the decoder of VGAE calculates cell-cell similarity between by calculating the dot product between latent representations of cells to generate the CCI network.

Spatial Variance Component Analysis models CCI based on the assumption that CCI is a cause of cellular variation, and gene expression variance in cells can be modeled as a linear combination of three components: an intrinsic cell state effect, an environmental effect accounting for the position of the cell, and a cell-cell interaction effect [48]. The three variational effects are then modeled as multivariate Gaussian distributions whose covariance matrices account for similarity in cell intrinsic state, spatial proximity, and cellular neighborhoods. The covariance matrix that account for similarity of cellular neighborhood is the term of interest that can account for cell-cell interactions, and it is determined by fitting a regression model using maximum likelihood estimation for each target gene.

### 2.6. Significance test

Some CCI tools incorporate a significance test, including permutation test and Kendall’s rank-correlation coefficient, on the calculated CCI scores to filter out non-significant interactions. One common method used is the permutation test. After calculating a CCI score of a pair of ligand-receptor from sender to receiver cell types, the cell type labels are randomly permuted for a large number of times to generate a null distribution. The *p*-value of an interaction is calculated as the probability of obtaining a CCI score that is higher than the original inferred CCI score. Only CCI scores with corresponding *p*-values that are lower than a specified threshold are kept. The procedures are very similar to permutation based (cell type label permutation) intercellular CCI calculations, though in significance tests they are performed with a different purpose, which is to filter out nonsignificant interactions. Permutation tests are used in CellChat [19] and Graeber and Eisenberg [49]. Some methods, such as PyMINER [21], use an ANOVA test to calculate a *p*-value, using to determine differentially expressed ligand and receptor genes. Other methods, such as scConnect [31], PyMINEr [21], and ICELLNET [50], correct for multiple testing via, for example, the Benjamini-Hochberg correction. In contrast, Kendall’s rank-correlation coefficient is used in scMLnet [51] to filter out weakly correlated links from receptors to transcription factors and links from transcription factors to downstream target genes [38]. To infer links between receptor genes and transcription factors, the gene expression values receiver cells are used to compute the Kendall’s rank-correlation coefficient between receptor genes and transcription factors. Links with a Kendall’s rank-correlation coefficient below a specified threshold are removed. A similar method is used to remove links between transcription factors and target genes.

## 3. B. downstream analysis of CCI

After infering CCI, several methods go further and extract features based on the properties of the CCI networks, including network centrality analysis [52,34,35], network diversity analysis [53], gene entropy analysis [26], communication pattern analysis [52], classification of signaling pathways [52], and comparison between multiple datasets [40,28,52,34,53,22]. In spatial methods, downstream analysis include visualization of the signaling direction in space, and identification of differentially expressed genes affected by CCI [45].

CCI networks can be represented as a directed weighted network, where the nodes represent cell groups and the edges represent directed interactions from sender cell groups to inferred receiver cell groups. Therefore, many CCI downstream analysis methods exploit these network-level features of CCI networks, including network centrality analysis, network diversity analysis, functional and structural similarity of CCI networks [52]. Network centrality, especially node centrality, is used to discover important senders, receivers, mediators and influencers in the CCI networks. The in-degree and out-degree measure the total interaction strength received and sent by a cell group, respectively. Flow betweenness measures the implied ability of a cell group to control the interaction flow between different cell groups. Information centrality provides the amount of control on the information flow. Many other popular centrality measures, including hub score, authority score, EigenCentrality, and PageRank can also help identify important cell groups in CCIs [52]. In addition, network diversity analysis can extract node-level, node-to-node-level, and network-level diversity of multiple CCI networks that represent the CCIs for different biological conditions [53]. For example, node degree centrality has been used to analyze COVID-19 datasets, where it has been found that there is a decreasing trend of cell interactions received by B cells as the severity of COVID-19 increases [53]. Significant signaling pathways present in the CCI networks can be classified according to pairwise functional similarity and structural similarity, where functional similarity quantifies the similarity of major senders and receivers in each signaling network, and structural similarity measures the topological differences between signaling networks [52]. Non-negative matrix factorization is used to identify how sender cells and receiver cells coordinate with each other with respect to the signaling pathways they use [52]. Apart from network analysis, gene entropy analysis measures the amount of uncertainty in the signaling from a specific gene to another [26]. It is based on an inferred gene-gene network, where each node indicates a gene and each edge indicates the interaction strength between the two genes. The signaling entropy of a gene in the network represents the uncertainty in the signaling activity to other genes, and is calculated using Shannon entropy [54].

Comparison of CCI features between multiple datasets is another popular downstream analysis. For example, CellChat implements methods to compare differences in interaction strengths, major sender and receiver cell groups, interaction strengths in signaling pathways, and upregulated and downregulated ligand-receptor pairs between conditions [52]. Network diversity analysis also enables the comparison of differences in CCI diversity across multiple conditions [53].

Among all spatial CCI methods, COMMOT performs some downstream analysis based on the inferred CCI [45]. The spatial vector field of signaling directions of incoming and outgoing cells are visualized in a spatial signaling direction plot. Also, differential gene analysis is performed to identify differentially expressed genes that are affected by the spatial CCI.

## 4. C. experimental validation

As many computational tools have been developed to measure CCI levels, it is important to validate the predicted interactions. There are different ways to validate the calculated CCI levels, including experimental methods, spatial colocalization and literature support.

Experimental validation can be performed in three different ways: (1) Validation of the expression of ligands and receptors involved in CCI using proteomics, enzyme-linked immunosorbent assay, immunohistochemistry or western blots. (2) Functional assessment of CCI roles by in vivo or in vitro experiments using activators or inhibitors of involved ligands and receptors. (3) Visualization of CCI between neighboring cells using microscopy and immunostaining, single-molecule fluorescence in situ hybridization, and flow cytometry [55].

Spatial colocalization validation assumes that spatially adjacent cells are more likely to interact than cells that are further apart. Thus, a reasonable CCI score should have the property that it is larger between adjacent cells and lower in further apart cells. Since this type of validation requires spatial information of cells, it is normally performed on spatial transcriptomics dataset.

Evidence from biological literature is also a popular method to indirectly validate the predicted CCIs. For example, when studying CCI between dermal condensate (DC) and epithelial placode cells in hair follicle morphogenesis, CellChat inferred that epithelial placode cells distinctly secrete *Fgf20* ligand to all DC states, which is consistent with the previously known role of placode-derived *Fgf20* signaling [52].

### 4.1. Analysis of CCI in cancer

Cancer is a current major health concern worldwide. Genetic and epigenetic alterations induce changes in cell-cell interactions that let cells escape homeostatic controls and drive cancer. Changes in CCI influence not only cancer cells but also other important components in the tumor microenvironment, including immune cells, endothelial cells, surrounding stromal cells, and others [56]. Ligand-receptor interactions between cancer cells and normal cells and intracellular signaling are capable of regulating multiple crucial cellular processes during the progression of cancer, including cell growth and division, cell apoptosis, cell motility and invasion, angiogenesis, inflammation and immune suppression [7–9]. For example, Sever and Brugge give a comprehensive examination of how changes in PI3K-Akt and Ras-ERK interactions to can cause different characteristic features of cancer to emerge [9]. Due to its clear importance in cancer, the analysis of CCI can potentially greatly enhance our understanding of cancer mechanisms. In pharmacology, targeting the interacting cells or mediators of cell interactions has been proven to be effective in multiple tumor treatments [9,39].

Using computational CCI tools, one can quantitatively study the upregulated or downregulated ligands and receptors within and between normal cells and tumor cells to potentially identify novel cell interactions, raise new hypothesis and provide evidence on cell interaction mechanisms in cancer development. Here, we highlight but a few applications. For example, Choi et al. used CCCExplorer [39] to identify new ligand-receptor interactions, including IL6-IL6R and WNT11-FZD7, between macrophages and tumor cells in non-small cell lung cancer (NSCLC). Choi et al. also verified the presence of AREG-EGFR signaling from monocytes to tumor cells, which has been reported in many other cancer types. Since AREG has been reported to inhibit apoptosis, the presence of this interaction suggests a potential tumor-promoting role of monocytes in NSCLC [39]. In another study, using human scRNA-seq data of head and neck squamous cell carcinoma (HNSCC) tumors, Browaeys et al. used NicheNet to investigate the hypothesis that cancer-associated fibroblasts (CAFs) may regulate genes involved in the partial epithelial-to-mesenchymal transition (p-EMT) program, as originally proposed by Puram et al. [40]. They inferred the top interactions between CAF-ligands and p-EMT target genes in cancer cells, including interactions from TGFB3 to TGFBI, LAMC2, and TNC, shedding further light on the potential regulatory roles of CAF-ligands on p-EMT target genes. Arnol et al. [48], the developers of SVCA, studied a breast cancer Imgaging Mass Cytometry (IMC) dataset. Using SVCA, they inferred that CCI components can explain up to 25% of the gene expression variance, where marker genes of immune cells, including CD44, CD20, CD3 and CD68, comprised the largest CCI effects. By using CellCall to study CCI between immune cells from six types of cancer, including liver hepatocellular carcinoma, non-Hodgkin lymphoma, non-small cell lung cancer, kidney renal clear cell carcinoma, colorectal cancer, and breast invasive carcinoma, Zhang et al. inferred that monocytes and macrophages receive the largest amount of signals from other immune cell types, indicating their roles as dominant signaling receivers among all immune cell types in multiple cancers [25].

Due to frequent mutations occurring in cancer, there are some limitations of using computational CCI tools to infer cell interactions in cancer. First, cancer-causing mutations may lead to distortions in inferred interactions. For example, a recent study has shown that in melanomas, lung and colon cancers, the gene BRAF mutation V600E will induce a novel interaction between BRAF and KEAP1 [57]. Such distorted interactions caused by mutations may not be captured by currently available CCI methods or databases. Furthermore, for methods that consider downstream effects of ligand-receptor interactions, if one of the downstream genes is mutated, this could affect the entire downstream network significantly, which may, in turn, affect CCI inference.

### 4.2. Future directions

With the vastly increasing number of scRNA-seq and spatial datasets and the rapidly developing technologies, opportunities arise as we try to increase our understanding of CCI and improve the reliability of CCI tools.

### 4.3. Incorporating spatial information

Incorporating spatial information has many advantages. First, cells primarily interact with each other locally and across limited spatial distances. While scRNA-seq provides the gene expression of ligands and receptors in cells, which is important for the detection of CCI, it does not capture the physical positions of cells, missing an important spatial constraint on possible interactions. Second, non-spatial data cannot accurately infer CCI that occur through physical cell-cell contact. It is possible that future work can model the cell-cell contact by incorporating cellular spatial information. Third, most current CCI methods calculate the interaction strengths between groups of cells to reduce false positive estimates. However, these methods implicitly assume that all cells within the sender and receiver groups are in signaling range. It may be the case that only a subset of these cells are close enough to interact. Thus, it is crucial to develop more tools that can map a spatial distance between cells, by either equipping spatial positions to non-spatial cells, or by using ST to profile the cells together with their spatial positions.

### 4.4. Incorporating temporal information

Most CCI methods rely on the average ligand and receptor expression in different groups of cells to infer CCI from scRNA-seq data. However, as scRNA-seq is a static snapshot of gene expression, these methods an important aspect of CCI, which is that it is dynamic. Incorporating time information provides a new angle of studying CCI in general. Apart from learning how CCI evolves with respect to time, time information may also aid in finding associations between CCI and key biological processes that may be important in cancer, including cell differentiation, organ development, disease progression, and immune response. To account for temporal information, one could analyze CCI across a time series dataset of scRNA-seq data sampled from the same biological system at multiple time points, or by applying a pseudotime ordering method on a single dataset and analyzing CCI across inferred pseudotime branches [19,58].

### 4.5. Competition and cooperation between CCI

Currently, most CCI tools rely on the assumption that ligand-receptor interactions occur are independently, which may not be the case. For example, researches have shown that the invasion of a human mammary tumor is driven by both paracrine signaling to and from host macrophages and autocrine signaling between tumor cells [59]. It may therefore be the case that these interactions co-interact. Furthermore, multiple ligands compete to bind to the same set of receptors, but current methods assume that these ligands can bind to the same receptors across all possible receiver cells. Thus, future work could incorporate the dependency between ligand-receptor interactions to improve accuracy of inferred CCI. There has been some recent research in this direction [35,60].

### 4.6. Multi-omics integration

The overwhelming majority of current CCI methods focus on intercellular signaling and do not account for the intracellular downstream response (with some exceptions). A key aspect of intracellular signaling is the activation or in activation of transcription factors after ligand-receptor binding. One example is the activation of transcription factors in the SMAD family due to TGF-β signaling [61]. Once a transcription factor is activated, it binds to specific regions of non-coding DNA to modulate the transcription downstream target genes as a response to cell signaling. Given their evidently important role in the intracellular signaling network, accounting for the signaling gene regulatory network facilitated by transcription factors can provide extra information when studying CCI. Such information can be obtained through several recently developed technologies. For example, advances in mass spectrometry have enabled the high-throughput measurements of post-translational modulation of signaling proteins [61]. Other technologies like single-cell Assay for Transposase-Accessible Chromatin using sequencing (scATAC-seq) and Chromatin immunoprecipitation followed by sequencing (ChiP-seq) provide information on chromatin structure and accessibility, which regulate binding affinity of transcription factors and their consequent regulation of downstream target genes [62]. The incorporation of measurement of transcription factor activity from other omics data is promising for both the inference and validation of CCI. Furthermore, as ligand-receptor interactions occur at the protein level, gene expression information alone cannot measure CCI directly. It is thus potentially promising to incorporate other technologies that provide protein level information. For example, cellular indexing of transcriptomes and epitopes by sequencing (CITE-seq) can integrate protein and transcriptome measurements into a single-cell readout [63].

## 5. Discussion

The development of both scRNA-seq and ST technologies has generated a new, rapidly expanding field of CCI studies. A wide range of computational tools have been developed recently to model and infer both intercellular and intracellular interactions in different species (e.g. human or mouse), and to analyze the downstream features of these constructed CCI networks. The currently available tools use different computational methods to model CCI from gene expression, based on their modeling assumptions. Even though ground truth datasets are currently lacking, some efforts have been made to compare between different CCI methods. For example, one study compares seven CCI methods with respect to: agreement of inferred CCIs with spatial colocalization, cytokine activities, and receptor protein abundance, concluding that the predicted CCI are coherent with these modalities in general [64]. They also found that different ligand-receptor databases used by different CCI methods have different biases towards certain pathways and tissue-enriched proteins. Another comparative study evaluates 16 CCI methods by integrating scRNA-seq with ST data, concluding that CellChat, CellPhoneDB, NicheNet, and ICELLNET show better performance in alignment with spatial information and in scalability [65]. For future work, we envision that CCI methods will be benchmarked based on their agreement with existing biological evidence. Though a large number of single-cell-focused tools have been developed, there are still limitations of current CCI inference methods. First, scRNA-seq data contain biological and technical noise in experimental processing [66], which may obscure the true gene expression of signaling genes. Second, ligand-receptor interactions occur at the protein level, meaning that the gene expression measurements provided by RNA-based measurements only give an indirect measurement of ligand and receptor expression. Third, most methods prioritize ligand-receptor interactions, but these are by no means the only form of CCI. For example, a recent method, NeuronChat [67] infers CCIs due to neurotransmitters consisting of groups of small molecules that are typically excluded from current ligand-receptor databases and corresponding receptors. Such interactions are not accounted for by other CCI methods and are known to play a role in glioma progression [68,69]. Fourth, while experimental validation, spatial colocalization, and literature support provide validation of the computed CCI scores from different tools to some extent, there is still a lack of ground truth datasets for which the underlying CCI networks are truly known, preventing substantial benchmarking of CCI inference methods. We propose that future work should try to improve the following aspects in developing computational tools to study CCI: (1) incorporate spatial information to improve the accuracy of calculated ligand-receptor scores; (2) incorporate temporal information to understand the dynamic process of CCI changes in time; (3) take into account the dependency of ligand-receptor interaction; (4) integrate scRNA-seq with other omics technologies to gain information at the protein and epigenetic levels and improve modeling and inference of CCI. With these points in mind, it is clear that the use of single-cell technology to analyze CCI will continue to grow at a rapid pace in the following decades to come, and so too will the insights that come with this growth.

## Figures and Tables

**Table 1:** Computational tools developed to infer CCI from non-spatial data.

Tool	Network type	CCI value	Method	Cancer Application	Visualization	Downstream pipelines	Platform

CellTalker	Intercellular	Bin.	Threshold	Head and neck cell carcinoma tumors	Chord diagram	None	R
PyMINEr	Intercellular	Cont.	Differential expression	None	Circle plot	None	Python
iTalk	Intercellular	Bin.	Differential expression	None	Network plot, chord diagram, errorbar plot	Differential CCI between datasets	R
CellPhoneDB	Intercellular	Cont.	Permutation	None	Dot plot, heatmap	None	Python
scSeqComm	Intercellular intracellular	Cont.	Permutation; Network; Fisher’s test	None	Chord diagram, heatmap, dot plot	None	R
scMLnet	Intercellular intracellular	Bin.	Differential Expression; Fisher’s test	None	Network plot	None	R/Python
CCCExplorer	Intercellular intracellular	Bin.	Differential Expression; Fisher’s test	Non-small-cell lung cancer	Network plot	None	software
CellCall	Intercellular intracellular	Cont.	Coexpression (sum-based); Differential expression	Testicular cancer	Chord diagram, Sankey plot	None	R
CytoTalk	Intercellular intracellular	Cont.	Coexpression (sum-based); Coexpression (correlation-based)	None	Network plot	Gene entropy analysis	R/Matlab
NicheNet	Intercellular intracellular	Cont.	Network	Head and neck cell carcinoma tumors	Chord diagram, heatmap	Differential CCI between datasets	R
CellChat	Intercellular	Cont.	Coexpression (product-based)	None	Hierarchy plot, circle plot, chord diagram, heatmap, bubble plot	1. CCI Network centrality 2. Communication patterns 4. Classify signaling pathways based on functional or structural similarity 5. Comparison analysis	R
NATMI	Intercellular	Cont.	Coexpression (product-based)	None	Heatmap, network plot, chord diagram	Differential CCI between datasets	Python
SingleCellSi gnalR	Intercellular	Cont.	Coexpression (product-based)	Melanoma; Head and neck cell carcinoma tumors	Chord diagram	None	R
ICELLNET	Intercellular	Cont.	Coexpression (product-based)	Breast cancer	Chord diagram, heatmap, bubble plot, barplot	None	R
CSOmap	Intercellular	Bin.	Coexpression (product-based)	None	None	None	Matlab
scConnect	Intercellular	Cont.	Coexpression (product-based)	Melanoma	Network plot, sankey plot, heatmap	Differential CCI between datasets	Python
soptSC	Intercellular	Cont.	Coexpression (product-based)	None	Chord diagram, network plot	None	R/MatLab
Connectome	Intercellular	Cont.	Coexpression (product-based)	None	Chord diagram	Network centrality analysis, differential CCI between datasets	R
REMI	Intercellular	Cont.	Coexpression (correlation-based)	Lung squamous cell carcinoma	Chord diagram, heatmap	Network centrality analysis	R

Under the column ‘CCI value’, cont. and bin. refer to continuous and binary, respectively.

**Table 2:** Computational tools developed to infer CCI from spatial transcriptomics.

Tool	Network type	CCI value	method	Cancer Application	Visualization	Downstream pipelines	Platform

SpaOTsc	Intercellular	Cont.	Optimal transport	None	None	None	Python
COMMOT	Intercellular	Cont.	Optimal transport	Breast cancer	None	Spatial signaling direction, genes regulated by signaling	Python
Giotto	Intercellular	Cont.	Coexpression (sum-based)	Triple negative breast cancer	Network plot, dot plot, heatmap	None	R
GCNG	Intercellular	Bin.	Graph convolutional network	None	None	None	Python
DeepLinc	Intercellular	Cont.	Variational graph autoencoder	Breast cancer	None	None	Python
SVCA	Unspecified	Cont.	Gaussian process model	Breast cancer	None	None	Python

Under the column ‘CCI value’, cont. and bin. refer to continuous and binary, respectively.

## Data Availability

No data was used for the research described in the article.
